# Trastuzumab-associated Posterior Reversible Encephalopathy Syndrome

**DOI:** 10.7759/cureus.2686

**Published:** 2018-05-24

**Authors:** Omar Abughanimeh, Mouhanna Abu Ghanimeh, Ayman Qasrawi, Laith A Al Momani, Sheshadri Madhusudhana

**Affiliations:** 1 Department of Internal Medicine, University of Missouri Kansas City School of Medicine, Kansas City, USA; 2 Department of Internal Medicine-Gastroenterology, Henry Ford Health System, DETROIT, USA; 3 Internal Medicine, East Tennessee State University; 4 Hematology and Oncology, University of Missouri Kansas City School of Medicine, Kansas City, USA

**Keywords:** posterior reversible encephalopathy syndrome, trastuzumab, gastric cancer

## Abstract

Posterior reversible encephalopathy syndrome (PRES) is a clinical-radiographic syndrome that presents with neurological manifestations, including seizures, headache, or confusion, and is associated with posterior cerebral white matter edema on imaging. PRES is typically a benign and reversible condition. However, PRES can be fatal or associated with permanent deficits. Numerous conditions are associated with PRES, including hypertensive encephalopathy, renal diseases, and cytotoxic or immunosuppressant drugs. Recently, many case reports described the association between PRES and chemotherapeutic agents. However, trastuzumab-associated PRES is rarely reported. Herein, we report a case of a 51-year-old female with a history of metastatic gastric cancer who developed seizures while being treated with trastuzumab, and neuroimaging confirmed the diagnosis of PRES.

## Introduction

Posterior reversible encephalopathy syndrome (PRES) is a clinical-radiographic syndrome that presents with seizures, altered mental status, or headaches and is associated with posterior cerebral white matter edema on imaging [1-2]. PRES is commonly associated with hypertensive encephalopathy, eclampsia, and immunosuppressive agents. Recently, chemotherapy and targeted agents were reported as another potential cause [3]. Two theories explain the pathogenesis of PRES: the vasogenic theory and the endothelial theory. Both theories involve an increased permeability of the blood-brain barrier (BBB), which leads to brain edema [4]. Magnetic resonance imaging (MRI) of the brain is the gold standard to diagnose PRES and exclude important differential diagnoses. The parieto-occipital regions are the most commonly affected, but any area of the brain can be involved [2]. Treatment of PRES is supportive. This syndrome is typically benign but can be fatal or associated with permanent neurological deficits [2].

## Case presentation

A 51-year-old female had a history of hypertension and stage 4, human epidermal growth factor receptor 2 (HER-2) positive, gastric adenocarcinoma with peritoneal, bone, and lymph nodes metastasis. She presented with an episode of a tonic-clonic seizure. She did not have any previous history of seizures. The patient was diagnosed with stage 4 cancer two years ago. She was started on trastuzumab, cisplatin, and capecitabine for three cycles and continued trastuzumab until her presentation (the last cycle was four weeks prior to presentation). Her vital signs and labs are presented in Table 1.

**Table 1:** Patient’s vital signs and laboratory workup.

Patient’s vital signs and laboratory workup	
Vital signs	
Blood pressure (mmHg)	160/96
Heart rate (bpm)	93
Respiratory rate (per minute)	18
Oxygen saturation (%)	99
Complete blood count	
Hemoglobin (g/dl)	11.6
White blood cells (/cmm)	11,700
Platelet count (/cmm)	199,000
Chemistry	
Sodium (mmol/L)	140
Potassium (mmol/L)	3.8
Magnesium (mmol/L)	1.2
Chloride (mmol/L)	100
Glucose (mg/dl)	136
Blood urea nitrogen (mg/dl)	21
Creatinine (mg/dl)	1.3
Bicarbonate (mmol/l)	21
Ethanol level	< 10
AST (Units/L)	25
ALT (Units/L)	24
ALP (Units/L)	56
Total bilirubin (mg/dl)	1.1

**Figure 1:**
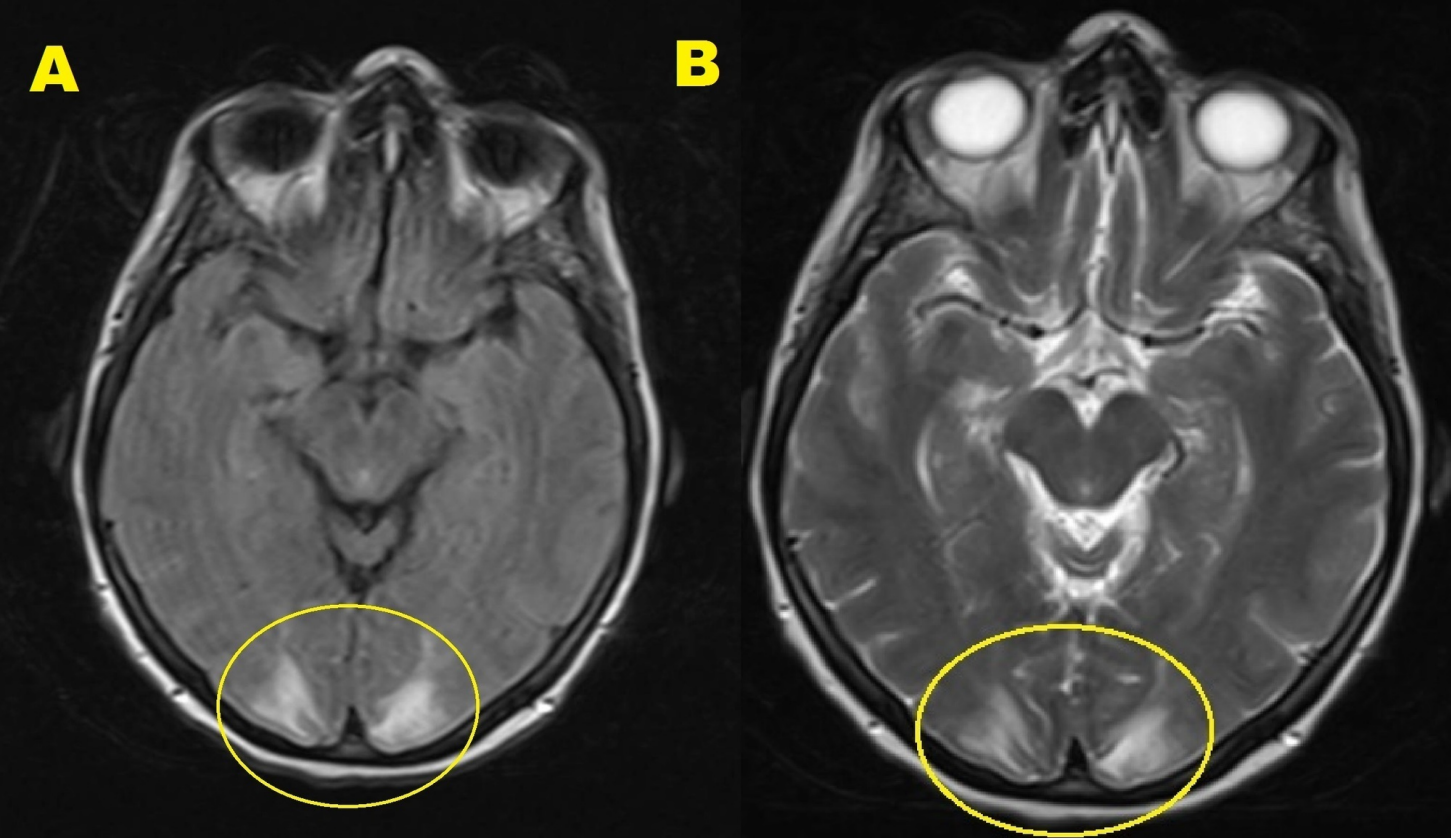
Brain MRI w/wo contrast. A) T2-TSE sequence, B) T2-FLAIR sequence. Showing bilateral, symmetric areas of T2-FLAIR hyperintensity involving the posterior parietal and occipital lobes. There was no evidence of acute stroke, intracranial hemorrhage, hydrocephalus, dural venous sinuses thrombosis, or intracranial metastatic disease. T2-TSE: T2-weighted turbo spin echo; T2-FLAIR: T2-weighted-fluid-attenuated inversion recovery.

**Figure 2:**
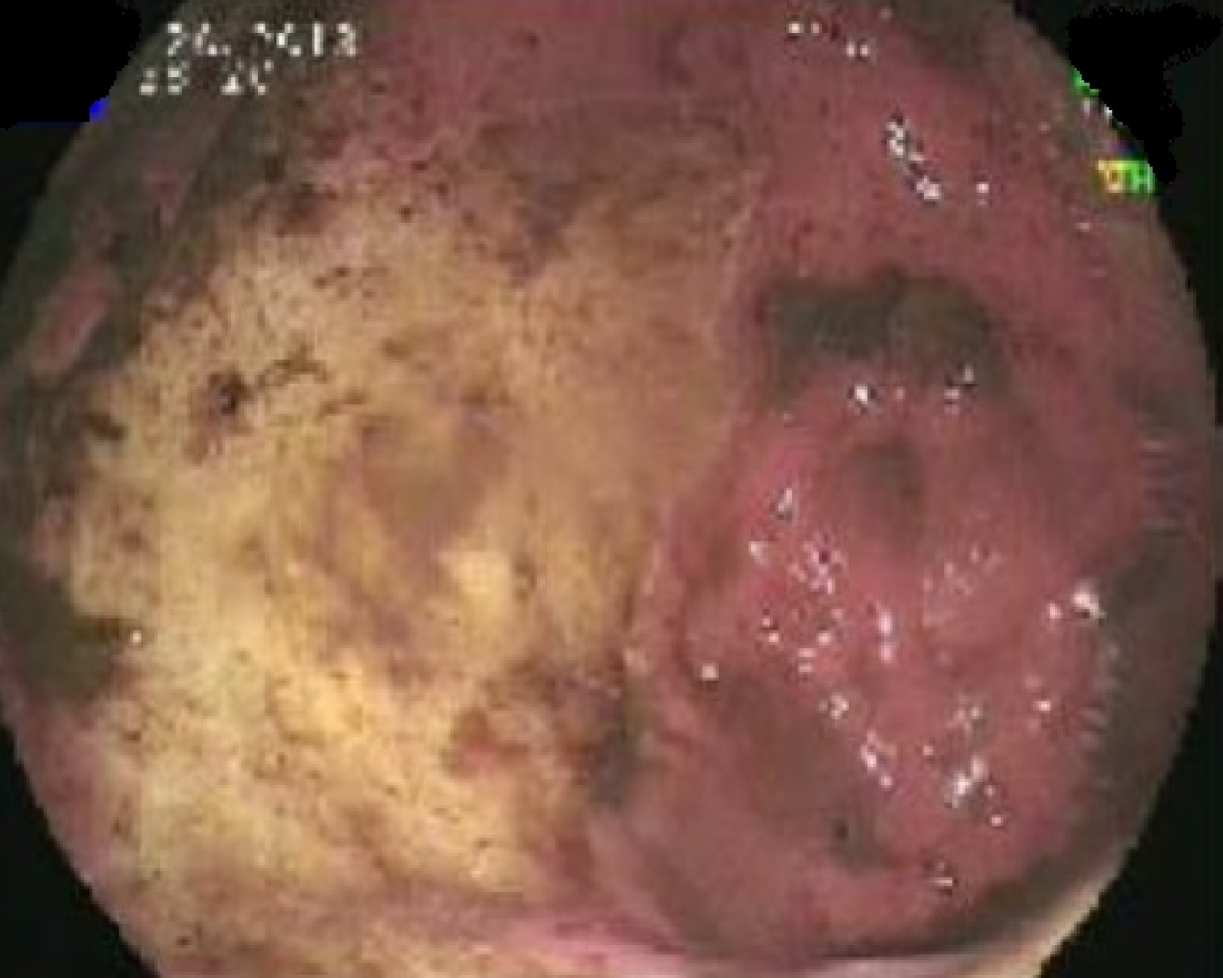
EGD showing a large malignant looking, friable, fungating, ulcerated mass in the gastric body. EGD: esophagogastroduodenoscopy.

A brain MRI with and without contrast (Figure 1) revealed bilateral, symmetric areas of T2-weighted-fluid-attenuated inversion recovery (T2-FLAIR) hyperintensity involving the occipital and posterior parietal lobes. There was no evidence of acute stroke, intracranial hemorrhage, or intracranial metastasis. These findings were suggestive of PRES. The multidisciplinary tumor board committee decided that PRES could be attributed to the toxic effect of trastuzumab or hypertension. The patient was discharged on levetiracetam with a plan to continue trastuzumab, strictly control blood pressure (BP), and repeat the MRI in three months. During that period, the patient’s blood pressure was controlled on multiple encounters with all readings < 140/90. A repeated brain MRI with and without contrast revealed persistent T2-FLAIR hyperintensity in the occipital and posterior parietal lobes suggestive of PRES. The tumor board decided to hold trastuzumab for one month. The patient was admitted with a worsening epigastric pain and concern for disease progression. Esophagogastroduodenoscopy (Figure 2) revealed a large, malignant-appearing, partially obstructing mass in the gastric body with no active bleeding.

## Discussion

PRES is a clinical-radiographic syndrome of seizures, headaches, visual disturbances, and confusion associated with brain white matter edema on neuroimaging. PRES was first described by Hinchey et al. in 1996 [1-2]. The term posterior reversible encephalopathy syndrome is a misnomer, as this syndrome is not always reversible and is not limited to the posterior portions of the brain [2]. It is believed that PRES is underdiagnosed, given its variable clinical presentations [4].

The exact pathogenesis of PRES is unclear. Two theories explain how PRES develops. The first one is the vasogenic theory, where a rapid increase in blood pressure can cause an abnormality in cerebral autoregulation, leading to hyperperfusion and breakdown of the BBB with increasing permeability [3-4]. The second theory is the endothelial theory where toxic damage to the BBB leads to increased BBB permeability, resulting in edema. The endothelial theory provides a better explanation for chemotherapy-associated PRES, including PRES caused by targeted agents [4]. Moreover, it is believed that some chemotherapeutic agents and targeted agents can increase tumor cell recognition, resulting in an inflammatory reaction and changes in BBB permeability [2]. It is worth mentioning that targeted agents with antiangiogenic activity, such as trastuzumab, can also cause significant hypertension [5].

PRES is typically associated with hypertensive encephalopathy, eclampsia, and immunosuppressive agents [1-3]. Tam et al. [6] specified three risk factors for PRES: fluid overload (>10% of baseline weight), increased blood pressure (>25% of baseline) and creatinine >1.8 mg/dl. They recommended early neuroimaging to evaluate any unexplained neurological change in the patient with these risk factors. Moreover, electrolyte abnormalities, such as hypomagnesemia, are also a risk factor for PRES [7].

Chemotherapy-associated PRES is not common. How et al. [8] performed a systematic review of 70 cases of PRES due to chemotherapy. Most of these cases presented in the first week of chemotherapy administration. According to this study, platinum-based agents (cisplatin, carboplatin, and oxaliplatin) were reported in 30 cases and represented agents most commonly associated with PRES. Trastuzumab was not reported in any case in that study. To our knowledge, this is the first case of PRES after trastuzumab alone and the third case of PRES after a chemotherapy regimen that included PRES. Table 2 summarizes PRES cases occurring after trastuzumab.

**Table 2:** Reported cases of PRES after trastuzumab in English literature. PRES - Posterior reversible encephalopathy syndrome.

Case/primary malignancy	Patient’s age/gender	Chemotherapy regimen	Presenting symptom	Involved brain area	Outcome
Kaneda et al. [5]/gastric cancer	54 years old/female	Cisplatin, capecitabine, and trastuzumab	Loss of vision	Occipital lobes	Resolution
Ladwa et al. [7]/breast cancer	64 years old/female	Docetaxel, carboplatin, and trastuzumab	Generalized seizure	Occipital lobes	Resolution
Abughanimeh et al. (our case)/gastric cancer	51 years old/female	Trastuzumab	Generalized seizure	Occipital and parietal lobes	Died

Singer et al. [3] performed a retrospective study where they reviewed all cases of PRES in their cancer center from January 1, 2005, to June 30, 2011. They identified 31 cases; 21 of these patients were females. They found that PRES was more common in solid tumors (71%) compared with hematologic or primary brain tumors (25% and 3%, respectively). They found that the average time of PRES presentation was 22 months after the diagnosis of cancer.

PRES presents with headaches, seizure, confusion, changes in vision, and motor signs. These symptoms are a result of acute encephalopathy due to white matter edema [1]. Singer et al. [3] found that confusion was the most common presentation in 71% of the patients followed by seizures (58%), headaches (48%), and visual disturbances (26%).

MRI of the brain is considered the gold standard to diagnose PRES. Brain MRI typically reveals bilateral posterior white-matter edema in cerebral hemispheres especially the parieto-occipital regions; however, any part of the brain can be affected [1-2,7]. Fugate et al. [9] studied 120 cases of PRES and found that the parieto-occipital lobe was the most commonly affected area (94% of cases) followed by the frontal lobes (77%), temporal lobes (64%), and cerebellum (53%) [9].

PRES is typically a benign and reversible condition with most cases returning to baseline in days to weeks [2,6]. However, some reported cases exhibited fatal outcomes or permanent neurological deficits [2]. Early diagnosis is crucial to prevent complications. Suppurative treatment, blood pressure control, and stopping inciting factors represent typical measures [2]. Interestingly, re-exposure to the chemotherapeutic agent that caused PRES might be safe in the context of close monitoring and blood pressure control [3].

Our case was presented at the World Congress of Gastroenterology/American College of Gastroenterology annual meeting in 2017 as a poster [Abughanimeh O, Abu Ghanimeh M, Qasrawi A, Bahaj W, Madhusudhana S. "Trastuzumab-Associated Posterior Reversible Encephalopathy Syndrome in a Patient With Metastatic Gastric Cancer (Abstract)". Am J Gastroenterol, 2017; 112: S1442–S1443. DOI: 10.1038/ajg.2017.326; https://www.eventscribe.com/2017/wcogacg2017/ajaxcalls/PosterInfo.asp?efp=S1lVTUxLQVozODMy&PosterID=115733&rnd=0.2637929]

## Conclusions

Chemotherapy-associated PRES is increasingly reported in the literature. The early diagnosis of PRES is crucial to prevent complications. Given the recent increased use of trastuzumab, physicians should consider PRES in cases of sudden, unexplained neurological manifestations in patients receiving it.
